# Gut microbiota and sleep: Interaction mechanisms and therapeutic prospects

**DOI:** 10.1515/biol-2022-0910

**Published:** 2024-07-18

**Authors:** Zhonghui Lin, Tao Jiang, Miaoling Chen, Xudong Ji, Yunsu Wang

**Affiliations:** Department of Neurology Medical, Xiamen Hospital of Traditional Chinese Medicine, Fujian, Xiamen, China; Department of Cardiology Medical, Xiamen Hospital of Traditional Chinese Medicine, Fujian, Xiamen, China; Jimsar County of Xinjiang Chinese Medicine Hospital, Xinjiang, Changji, China

**Keywords:** gut microbiota, sleep disorder, bidirectional relationship, mechanisms, therapeutic interventions, personalized medicine

## Abstract

Sleep is crucial for wellness, and emerging research reveals a profound connection to gut microbiota. This review explores the bidirectional relationship between gut microbiota and sleep, exploring the mechanisms involved and the therapeutic opportunities it presents. The gut–brain axis serves as a conduit for the crosstalk between gut microbiota and the central nervous system, with dysbiosis in the microbiota impairing sleep quality and *vice versa*. Diet, circadian rhythms, and immune modulation all play a part. Specific gut bacteria, like *Lactobacillus* and *Bifidobacterium*, enhance sleep through serotonin and gamma-aminobutyric acid production, exemplifying direct microbiome influence. Conversely, sleep deprivation reduces beneficial bacteria, exacerbating dysbiosis. Probiotics, prebiotics, postbiotics, and fecal transplants show therapeutic potential, backed by animal and human research, yet require further study on safety and long-term effects. Unraveling this intricate link paves the way for tailored sleep therapies, utilizing microbiome manipulation to improve sleep and health. Accelerated research is essential to fully tap into this promising field for sleep disorder management.

## 1 Introduction

Sleep, an essential physiological process, is now recognized as a crucial regulator of our gut microbiome – the intricate ecosystem residing within us, demonstrating an unexpected yet profound interplay between microscopic inhabitants and our sleep. Disruptions in sleep patterns, such as insomnia or sleep deprivation, not only impair cognitive function and emotional wellbeing [1,2] but also trigger a cascade of biological perturbations that reverberate throughout the body [3,4], particularly affecting the delicate balance of the gut microbiota [5]. This intimate link poses intriguing questions about the potential of modulating gut microflora as a therapeutic avenue for mitigating sleep disorders.

The gut microbiota, a complex consortium dominated by bacteria from phyla Firmicutes, Bacteroidetes, and others, operates in a delicate harmony vital for maintaining homeostasis [6]. This equilibrium, however, is fragile and easily upset by lifestyle factors, including sleep habits, diet, and stress, among others [7–9]. The gut–brain axis, a bidirectional communication network, facilitates the microbiota’s influence on host physiology, including sleep regulation, underscoring the microbiome’s centrality in holistic health.

Recent scientific inquiries have uncovered a reciprocal relationship: while disrupted sleep patterns can disturb the gut microbiota, leading to dysbiosis and subsequent health implications, an imbalance in the microbiome can also exacerbate sleep issues, perpetuating a detrimental cycle [10–12]. This reciprocal symbiosis highlights the criticality of a balanced gut microbiome to overall health and underscores sleep as a pivotal modulator.

Given the established correlations and the potential for translational applications, exploring the precise mechanisms governing the gut microbiota-sleep axis becomes imperative. This review synthesizes the latest advancements in our understanding of the intricate dance between sleep disorders and the gut microbiome, aiming to distill the essence of this complex relationship. By illuminating these pathways, we strive to pave the way for targeted interventions in sleep medicine, emphasizing the potential of gut microbiota manipulation as a promising frontier in addressing sleep disturbances. Understanding these dynamics is not merely academic; it holds the key to transforming clinical practices, offering hope for novel, microbiome-centered therapies to restore sleep health and, consequently, enhance overall wellbeing.

## 2 The impact of sleep disorders on the gut microbiota

The composition of the gut microbiota is host-specific, shifting over an individual’s lifespan and readily susceptible to both environmental and intrinsic factors. The changes in gut microbiota exhibit clear circadian rhythms. Accumulating scientific evidence underscores a profound interconnection between the oscillations of human sleep-wake cycles and the gut’s microbial communities. Various sleep disorders have been shown to disrupt the circadian rhythms of gut microbiota, leading to ecological imbalances (Table 1), which, in turn, exacerbate sleep problems and contribute to the development of complications, including chronic inflammation, cardiovascular, and cerebrovascular diseases.

**Table 1:** An overview of studies examining gut microbiota changes in sleep disorders

**Models**	**Subjects**	**Sample size**	**Major findings of gut microbiota**	**Reference**
**Disruption of circadian rhythms**				
Jet-lag	C57Bl/6 mice (8–9 weeks old)	10 mice/group	Increase in the *Paraprevotella*, *Fusobacteria*, and *Fusobacteriales*. Decrease of the *Christensenellaceae*, *Dorea*, *Anaeroplasmatales*, *Anaeroplasmataceae*, *Anaeroplasma*, *Lactobacillus*, *Lactococcus*, *RF32*, *Alphaproteobacteria*, *Proteobacteria*, *Ruminococcus*, and *Ligilactobacillus ruminis*. Promotes glucose intolerance and obesity	[14]
Jet-lag	C57BL/6J mice (8–10 weeks old)	6 mice/group	Decreased in the microbial abundance, richness, and diversity	[16]
			Decreased in the Bacteroidetes phylum	
			Increased in the F/B ratio and Actinobacteria phylum	
			Decreased in the levels of tryptophan and its derivatives in fecal samples	
Sleep–wake cycle shift	Human beings (aged 20–35 years)	22 volunteers	Increased in the F/B ratio	[17]
			Increased in the phyla Fusobacteria and Tenericutes and classes	
			Fusobacteriia and Mollicutes. Decreased in the order Pasteurellales and family Clostridiales, Peptostreptococcacea. Changed in the functional profile of gut microbiota Changed in the microbial networks	
**OSAS**				
OSAS	C57BL/6 mice (6 weeks old)	8 mice/group	Increased in the α-diversity and β-diversity	[20]
			Enrichment in the Firmicutes	
			Decreased in the Bacteroidetes and Proteobacteria phyla	
OSAS	Human beings (aged 2–12 years)	16 children (7 OSASs and 8 controls)	Decreased in the gut microbial diversity	[21]
			Increased in the *Bacteroides fragilis*	
			Pro-inflammatory strains (including *Proteobacteria*, *Clostridiaceae*, *Oscillospiraceae*, and *Klebsiella*) are increased	
OSAS, hypertension (HTN)	Human beings (adults)	52 (9 controls, 17 OSAS only, 5 HTN only, and 21 OSAS + HTN)	Increased in the F/B ratio	[22]
			OSAS had higher *Ruminococcus_1*, *Lachnoclostridium*, *Lachnospira*, *Ruminococcus_torques*_group, and unidentified *Lachnospiraceae* levels than those without OSA	
			In OSA patients, hypertensive patients had lower *Faecalibacterium* and *Lachnospiraceae*_NK4A136_group levels	
OSAS	Human beings (adults)	113 volunteers (93 OSASs and 20 controls)	Decreased in SCFA-producing bacteria and increased in pathogens	[23]
			Decreased in *Megamonas*, *Ruminococcaceae*, *Alistipes*, *Dialister*	
			*Oscillibacter*	
			Elevated levels of IL-6	
OSAS	Human beings (adults)	48 (37 OSAS and 11 control)	Enriched *Fusobacterium*, *Megamonas*, and *Lachnospiraceae* UCG_006 and reduced *Anaerostipes* in patients with severe OSA	[24]
**Sleep fragmentation**				
Sleep fragmentation	C57BL/6J mice (8 weeks old)	60 (30 mice/group)	Increased of food intake	[25]
			Enrichment of highly fermentative members of *Lachnospiraceae* and *Ruminococcaceae*	
			Decreased in *Lactobacillaceae* families	
Acute sleep deprivation	C57BL/6J mice (age: 7 weeks)	20 (10 mice/group)	Increased in food intake, yet decreased in the body weight	[26]
			Decreased in the αand β-diversity of gut microbiota	
			Increased of the *Candidatus_Arthromitus* and *Enterobacter*	
			Decreased in the *Lactobacillus*	
			*Muribaculum*, *Monoglobus*, *Parasutterella*	
Sleep fragmentation	Sprague-Dawley rats (4 weeks old)	9–12 mice/group	Significant perturbations in alpha- and beta-diversity	[27]
			**Acute SF:** decrease in the *Lactobacillaceae*, *Lachnospiraceae*, F16, and *Alcaligenaceae* families; increase in S24-7 and *Porphyromonadaceae* families in the distal ileum	
			**Chronic SF:** decrease in *Enterobacteriaceae* and *Lactobacillaceae* Families; increase in *Turicibacteraceae* and *Clostridiaceae* families	
Sleep deprivation	C57BL/6J mice (age: 8 weeks)	30 (10 mice/group)	Increased of Tannerellaceae, Rhodospirillales, Alistipes, and Parabacteroides in SD mice	[28]
Sleep deprivation	Humans	25 volunteers	Decreased in the a-diversity of gut microbiota	[29]
			Decreased of *g_Prevotella*, *g_Sutterella*, *g_Parasutterella*, *g_Alloprevotella*, *g_Anaeroplasma*, and *g_Elusimicrobium*	
			Decreased of the concentration of the SCFAs (including acetate, propionate, and butyrate) in fecal samples	
**Narcolepsy**				
Narcolepsy type 1 (NT1)	Humans	35 patients and 41 controls	No between-group differences for alpha diversity	[30]
			Decreased in *Bacteroidetes*, *Bacteroides*, and increased of *Flavonifractor*	
NT1	Humans	20 NT1 patients and 16 controls	No differences in alpha and beta diversity were observed between the two groups	[31]
			Enrichment of the *Klebsiella*	
			Decreased in the *Blautia*, *Barnesiellaceae*, *Barnesiella*, *Phocea*, *Lactococcus*, *Coriobacteriia*, *Coriobacteriales*, *Ruminiclostridium*_5, and *Bilophila*	
**Insomnia**				
Acute and chronic insomnia	Humans	20 acute insomnia (AID), 38 chronic insomnia (CID), and 38 healthy controls (HC)	**Insomnia patients vs HC:** lower microbial richness and diversity, depletion of anaerobes, and SCFA-producing bacteria, and an expansion of potential pathobionts.	[32]
			**AID vs HC:** decreased abundance in *Faecalibacterium*, *Prevotella* 9, and *Roseburia*, and increased abundance in *Blautia* and *Eubacterium hallii.*	
			**CID vs HC:** increased abundance in *Bacteroides* and decreased abundance in *Lachnospira*.	

### 2.1 Disruption of circadian rhythms

Maintaining a normal circadian rhythm is vital to individual physical and mental well-being, and circadian rhythm disruption is a significant risk factor for diseases such as obesity and type 2 diabetes [13]. Observations in the normal human body have revealed that over 60% of gut microbiota composition adheres to oscillatory rhythms, with approximately 20% of mouse gut commensals and 10% of human gut commensals demonstrating clear circadian variability [14]. Species in the human gut microbiota that exhibit robust rhythmicity primarily include *Parabacteroides*, *Lachnospira*, *Bulleida*, *Roseburia*, *Veillonella*, *Haemophilus*, *Adlercreutzia*, *Eggerthella*, *Anaerotruncus*, *Oscillospira*, *Ruminococcus*, *Holdemania*, *Desulfovibrio*, *Escherichia*, unspecified genera of families S24-7, and Enterobacteriaceae [14,15].

Circadian rhythm perturbations trigger compositional shifts in the gut microbiome, marked by an increased Firmicute/Bacteroidetes ratio, depletion of *Christensenellaceae*, *Dorea*, *Anaeroplasmatales*, *Anaeroplasmataceae*, *Anaeroplasma*, *Lactobacillus*, *Lactococcus*, RF32, *Alphaproteobacteria*, *Proteobacteria*, *Ruminococcus*, and *Ligilactobacillus ruminis*, alongside enrichment of *Paraprevotella*, *Fusobacteria*, and *Fusobacteriales* [14,16,17]. Mouse models undergoing transplantation experiments have shown that circadian rhythm disruption alters gut microbiota profiles, causing weight gain and impaired glucose tolerance in mice [14], thereby implicating gut microbiota imbalance as a pivotal contributor to obesity development. These findings indicate that circadian rhythm disruption on gut microbiota dynamics and its implications for metabolic health.

### 2.2 Obstructive sleep apnea syndrome (OSAS)

Sleep apnea, a condition characterized by interrupted or shallow breathing during sleep, results in excessive daytime sleepiness and fatigue and poses a substantial risk for cardiovascular diseases, notably hypertension and stroke [18,19]. Studies in mice have shown that sleep apnea leads to a reduction in gut microbiota diversity and a shift towards a high Firmicutes/Bacteroidetes ratio [20]. In a pediatric cohort focusing on OSAS, Valentini et al. documented an enrichment of pro-inflammatory bacteria – Proteobacteria, Clostridiaceae, *Oscillospiraceae*, and *Klebsiella*, which impairs intestinal barrier integrity [21], further highlighting the detrimental effects on gut health. Hypertension, a frequent comorbidity with sleep apnea has also been linked to gut microbiota alterations. A cohort study involving 52 individuals, including 9 controls, 17 OSAS only, 5 hypertension only, and 21 suffering from both OSAS and hypertension, uncovered a distinctive increase in Ruminococcaceae and Lachnospiraceae genera among OSAS patients [22]. Collectively, these findings emphasize the alterations in gut microbiota composition in sleep apnea patients, which likely contributes significantly to the pathology of the condition.

### 2.3 Sleep fragmentation

Sleep fragmentation not only undermines health, cognitive performance, and physical prowess but also emerges as a significant risk factor in the development of neurodegenerative illnesses, such as Alzheimer’s disease [33]. Mouse models have shown that sleep fragmentation increases appetite and enhances the abundance of bacteria such as *Lachnospiraceae* and *Ruminococcaceae*, concurrently diminishing populations of *Lactobacillaceae* and *Bifidobacteriaceae* [25]. Studies spanning human and murine models highlight the anti-obesity and anti-inflammatory capacities of various *Bifidobacterium* strains [34,35]. In a study employing sleep-deprived C57BL/6 mice, subjects exhibited increased appetite alongside unexpectedly reduced body mass, coupled with profound disruptions to gut microbiota diversity, typified by an overrepresentation of *Candidatus_Arthromitus* and *Enterobacter*, and a depletion of *Lactobacillus*, *Muribaculum*, *Monoglobus*, *Parasutterella*, and other species [26]. Human studies parallel these findings, documenting declines in genera such as *Prevotella*, *Sutterella*, *Parasutterella*, *Alloprevotella*, *Anaeroplasma*, and *Elusimicrobium*, alongside reduced levels of acetate, propionate, and butyrate [29]. Collectively, these observations underscore the profound impact of sleep disturbances on the delicate microbial ecosystem equilibrium in both mice and humans, highlighting a crucial axis for future therapeutic interventions.

### 2.4 Narcolepsy

Narcolepsy, affecting roughly 0.02% of the global adult population, manifests through symptoms including excessive daytime sleepiness, cataplexy (sudden muscle weakness), sleep onset hallucinations, sleep paralysis, and disrupted nocturnal rest. This condition is further complicated by associations with psychiatric, cardiovascular, autonomic, and metabolic disorders [36,37]. In examining Type 1 Narcolepsy, two studies did not find differences in gut microbiota diversity measures (alpha and beta diversity) between the affected and healthy groups. However, they reported a conspicuous overabundance of *Klebsiella* genus and a marked decrease in the abundance of *Blautia*, *Barnesiellaceae*, *Barnesiella*, *Phocea*, *Lactococcus*, *Coriobacteriia*, and *Coriobacteriales* [30,31]. Mendelian randomization analysis have suggested that heightened levels of genus *Alloprevotella* and *Ruminiclostridium* 6 could potentially elevate the risk of NT1 [38]. These shifts in gut microbiota composition emphasize the substantial environmental influence in the development and pathophysiology of Type 1 Narcolepsy, pointing to potential environmental targets for further investigation and intervention strategies.

### 2.5 Insomnia

Insomnia, a condition marked by persistent sleep difficulties, has been found to be intricately connected to shifts in gut microbiota composition and heightened inflammatory responses, potentially unraveling new dimensions in our understanding and treatment of sleep disorders. A case–control study recruited 20 acute, 38 chronic insomnia, and 38 healthy controls and investigated gut microbiota and inflammatory cytokine changes in acute and chronic insomnia patients, revealing distinct microbial signatures correlated with sleep quality and elevated IL-1β [32]. Particularly, there is a conspicuous reduction in the populations of short-chain fatty acid (SCFA)-producing bacterial genera, notably *Roseburia* and *Faecalibacterium*, among individuals with insomnia [32]. These genera are recognized for their anti-inflammatory properties and positive contributions to health [39]. In rats, it has been demonstrated that chronic sleep restriction can increase pro-inflammatory cytokines, including IL-1β and TNF-α [40], whereas enhancing the variety of gut microbiota may inversely decrease circulating inflammatory cytokines, potentially leading to better sleep quality [41]. The results imply a complex interplay between insomnia, gut microbiome alterations, and inflammation, highlighting the microbiota’s potential as a diagnostic aid and therapeutic focus for sleep disorders.

## 3 Potential bidirectional interaction mechanisms between gut microbiota and sleep

**Figure 1:**
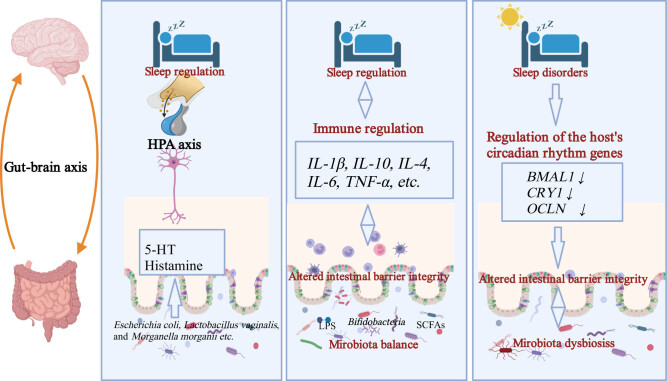
Schematic representation of potential bidirectional interaction mechanisms between gut microbiota and sleep. The bidirectional interaction between gut microbiota and sleep centered around the gut–brain axis encompasses three fundamental pathways: [1] Direct neurotransmitter influence: Gut microbiota-derived neurotransmitters like 5-HT and histamine directly affect the brain via the HPA axis, regulating the host’s sleep status. [2] Immune modulation: Gut microbiota and their metabolites stimulate immune cells such as dendritic cells, T cells, and B cells, modulating the immune state, which subsequently influences the host’s sleep patterns. Conversely, sleep disorders can lead to changes in immune status, resulting in gut microbiota dysbiosis [3]. Circadian rhythm and intestinal barrier integrity: Sleep disturbances induce alterations in the expression of circadian rhythm-related genes, disrupting the integrity of the intestinal epithelial barrier, ultimately leading to gut microbiota dysbiosis.

The intricate and reciprocal interplay between gut microbiota and sleep, with its potential for bidirectional influence, constitutes a complex yet rapidly evolving frontier of research in contemporary science. Growing evidence suggests that the microbiota–gut–brain axis plays a pivotal role in governing sleep behaviors through both direct and indirect mechanisms, with implications for the underlying causes and development of sleep disorders. Specifically, it has been documented that insufficient sleep induces disruptions in gut microbiota function, while the presence of sleep disorders coincides with marked shifts in the composition of the gut microbiota. Three pivotal aspects underpin the interactions between gut microbiota and sleep: first, microbial metabolites exert direct effects on sleep patterns through the gut–brain axis. Second, the microbiota modulates immune responses, indirectly impacting sleep regulation. Lastly, sleep and gene expression dynamics shape the composition of the gut microbiota (Figure 1).

### 3.1 The impact of gut microbiota metabolites on sleep

#### 3.1.1 Serotonin

Serotonin (5-hydroxytryptamine [5-HT]) is a monoamine neurotransmitter synthesized from the essential amino acid tryptophan has emerged as a key player in the microbiota–gut–brain axis, crucial in regulating emotions, hunger, pain processing, and sleep, alongside its role in gut motility and secretion [42]. Over 90% of bodily 5-HT is synthesized and released by gut enterochromaffin cells [43]. Studies in rats have shown that supplementing *Lacticaseibacillus rhamnosus* in rats significantly boosts 5-HT levels [44]. Key players in this gut–brain axis are bacteria from families such as Clostridiaceae and Turicibacteraceae, capable of producing and releasing 5-HT [45], reinforcing the gut microbiota’s integral role in 5-HT biosynthesis. The vagus nerve serves as a vital conduit connecting the gut’s activities to the central nervous system, facilitating communication via 5-HT receptors along its pathway. This neural link enables 5-HT to transmit signals to the brain, thereby regulating various physiological processes, including mood, with serotonin reuptake inhibitors’ antidepressant efficacy hinging on intact vagus nerve functionality [46]. Furthermore, chronic vagus nerve stimulation in cats leads to increased rapid eye movement (REM) sleep [47], underscoring the nerve’s impact on sleep patterns. Supplementation with *Ganoderma lucidum* in mice not only increased hypothalamic 5-HT concentrations but also ameliorated sleep quality [48], pointing to the potential of 5-HT modulation as a therapeutic target. Collectively, these findings suggest that manipulating gut microbiota to influence 5-HT levels presents a promising avenue for improving sleep quality, warranting deeper investigation in future research.

#### 3.1.2 Histamine

Histamine, functioning dually as a neurotransmitter and a signaling molecule, plays a pivotal role in regulating intestinal physiological functions, local immune responses, inflammation, and sleep–week cycle [49]. Of note, specific bacterial species, including *Escherichia coli*, *Limosilactobacillus vaginalis*, and *Morganella morganii* strains, possess the capability to both synthesize and release histamine [50]. The therapeutic arena witnesses the exploration of histamine H1 receptor antagonists, such as LY2624803, in ongoing clinical trials targeting insomnia [51], highlighting the clinical relevance of histamine modulation in sleep disorder management. This pharmacological intervention underscores the potential of gut-derived histamine as a key mediator in the gut–brain axis, influencing sleep quality. Unraveling the exact mechanisms by which microbial-origin histamine shapes sleep patterns necessitates rigorous, multidisciplinary research, aimed at clarifying the complex interplay between microbial activity, neural signaling, and circadian regulation.

#### 3.1.3 SCFA

Dietary fiber undergoes microbial metabolism in the gut, prominently including lactic acid bacteria (LAB) alongside genera such as *Ruminococcus*, *Lachnospira*, *Lachnospiraceae*, and *Ruminococcus* [52]. This metabolic process yields SCFAs, which are vital to maintaining intestinal homeostasis [53]. LAB contribute significantly to this process, fostering the production of SCFAs that reinforce the intestinal barrier, suppress oxidative stress, dampen inflammation, and preserve a stable gut ecosystem [52,53].

Sleep disturbances, however, disrupt this delicate balance by depleting populations of beneficial bacteria like LAB, thereby reducing SCFA levels. This decline in SCFAs is implicated in triggering neuroinflammation, a key factor affecting sleep quality [29]. Investigations into infant populations have revealed a positive correlation between a higher fecal content of propionic acid – a prominent SCFA – and prolonged periods of uninterrupted sleep in infants [54], implying a contributory role of SCFAs in determining sleep duration. The intricate relationship between sleep and inflammation is further illuminated by butyrate, a gut microbiota-derived metabolite known for its potent anti-inflammatory effects. Butyrate mitigates chronic inflammation, a frequent comorbidity with sleep disorders, by suppressing pro-inflammatory cytokine synthesis and inhibiting NF-κB activation [55,56]. This dual mechanism not only alleviates intestinal mucosal injury triggered by sleep deprivation but also fosters improved sleep quality. Additionally, studies have demonstrated that 72-h REM sleep deprivation can disrupt colonic circadian rhythms, accompanied by a decrease in SCFAs (propionic acid), intestinal inflammation, and enhanced intestinal permeability, resulting in disrupted circadian rhythms and gut microbiota dysbiosis [26], underscoring the delicate interdependence between sleep, gut health, and circadian homeostasis. In conclusion, these findings accentuate the critical role of LAB and their metabolites, especially SCFAs, in regulating sleep quality. They underscore the need for deeper mechanistic explorations to fully comprehend how these bacterial elements interact with sleep physiology, thereby informing potential therapeutic interventions for sleep disorders and gut health management.

#### 3.1.4 Lipopolysaccharide (LPS)

LPS, a key constituent of the Gram-negative bacterial outer membrane, typically exists at trace concentrations in the bloodstream in health status. However, heightened intestinal permeability can permit the translocation of LPS and other gut microbiota products into the circulation. Rodent studies have indicated that LPS administration prolongs non-REM sleep, with the process potentially mediated by IL-6 [57]. Microglia play a pivotal role in maintaining brain homeostasis and immune function. They have been shown to govern the sleep-wake cycle by modulating the anterior thalamic reticular nucleus neuron dynamics through ceramide-mediated signaling [58]. When LPS breaches the blood-brain barrier, it interacts with Toll-like receptor 4 (TLR4) on microglia, triggering a cascade of pro-inflammatory cytokine production. This inflammatory response can impose adverse hippocampal neurons [26,59,60], and alterations in microglial cell activity induced by LPS may contribute to the onset of neurological and sleep problems [60]. These findings collectively propose that LPS primarily modulates sleep status through its inflammatory response induction. Thus, restricting the overgrowth of LPS-producing bacteria emerges as a prospective strategy for managing sleep disturbances by mitigating inflammation and preserving the integrity of the gut–blood–brain axis.

#### 3.1.5 Other gut microbiota-associated metabolites

Studies have illuminated the pivotal role of neuroregulatory elements, including γ-aminobutyric acid (GABA), melatonin, and dopamine, as mediators through which the gut microbiome engages in sleep modulation. Melatonin, for instance, has demonstrated efficacy in reversing cognitive impairments and rectifying gut microflora imbalances precipitated by sleep loss. This restoration occurs via fine-tuning specific microbial populations and their metabolic byproducts, thereby attenuating hippocampal inflammation and neuronal apoptosis via intricate crosstalk between the TLR4/NF-κB and MCT1/HDAC3 signaling cascades, as reported in references [61,62]. In a rhesus monkey model, sleep deficiency has been linked to heightened stress hormone levels, systemic inflammation, and disruptions to the gut microbiota, implicating perturbations in GABA metabolism. Intervention with GABA-producing probiotics, notably *Lactobacillus* species, has proven efficacious in rehabilitating gut integrity and countering these adverse outcomes [63], implying that diminished GABA production in the gut could be central to the stress and gastrointestinal disturbances accompanying sleep deprivation.

### 3.2 The impact of gut microbiota on sleep through immunomodulation

The gastrointestinal tract, recognized as the largest immunological organ in humans, plays a crucial role in maintaining overall health [64]. Its dual function is paramount: first, maintaining the integrity of the intestinal barrier prevents deleterious microorganisms and their metabolites in the gut from entering the bloodstream, thereby preventing infections or systemic inflammation responses. Second, as a reservoir for a myriad of immune cells, including dendritic cells, macrophages, natural killer cells, and others. The gut fosters an environment where microbiota interact intimately with these cells. This interaction stimulates immune cell maturation and differentiation, preserving immune homeostasis and stabilizing the gut’s microbial ecosystem [64]. In a reciprocal dance, sleep and immune function are inextricably intertwined; immune activity exerts a regulatory influence on sleep patterns, while conversely, sleep quality impacts the immune system’s functionality and responsiveness [65]. This intricate balance highlights the interconnectedness of sleep, gut health, and immune competence in maintaining overall health and resilience.

Sleep disorder-induced immune dysfunction is a pivotal factor implicated in the pathogenesis of various diseases. Animal studies demonstrate that LPS, omnipresent in the gut, can induce inflammatory responses and damage the intestinal barrier by regulating the expression of TLR4 and NF-κB, thereby contributing to immune-related diseases induced by sleep deprivation [29]. Sleep deprivation results in a 1-4 fold decrease in anti-inflammatory cytokines, such as IL-4 and IL-10, concomitant with a surge (1- to 8-fold increase) in pro-inflammatory factors including IL-6, IL-1β, and TNF-α, disrupting the delicate balance. This also disrupts secondary bile acid metabolism, impairing colonization resistance mediated by gut microbiota [66]. Cohort analyses in humans reveal that insomnia can significantly elevate IL-6 levels compared to healthy individuals, with gut microbiota diversity positively correlated with both IL-6 levels and sleep duration [67,68]. *In vitro* supplementation of IL-6 in rats extends non-REM sleep duration [69]. Furthermore, reports underscore the intimate link between sleep quality and immune factors such as IL-1, IL-10, and TNF-β, emphasizing their regulatory roles [70,71]. Collectively, these observations underscore a bidirectional interplay between the gut microbiome and sleep, mediated through intricate immune regulatory mechanisms, highlighting the gut–sleep–health axis’s significance.

### 3.3 Potential interactions between gut microbiota and the expression of circadian genes

Sleep disruptions trigger multifaceted changes in the gut microbiota, affecting not only its structure but also functional capacity, which in turn feeds back to influence sleep quality. These alterations arise from the modulation of the host’s circadian rhythm genes, involving the downregulation of genes like brain and muscle ARNT-like 1 (BMAL1), cryptochrome circadian regulator 1 (CRY1), and occludin (OCLN). This disruption impairs the intestinal barrier’s integrity, diminishes the abundance of beneficial bacteria like lactobacilli and rumen ciliates, and fosters a state of gut microbiota dysbiosis, which consequently impacts sleep quality [26]. Notably, the central clock gene BMAL1’s dysfunction, as seen in knockout models, leads to compromised gut epithelial function, microbial imbalance, increased gut infection risk, and perturbed lipid metabolism [72], emphasizing the intricate link between circadian control and gut health.

Moreover, overexpression of the circadian rhythm gene CRY1 has demonstrated an ability to mitigate inflammation triggered by sleep deprivation [73], suggesting a direct role in sleep regulation. Conversely, sleep disorders can provoke intestinal mucosal damage alongside down-regulation of CRY1. Meanwhile, OCLN has been shown to mitigate inflammation and insulin resistance associated with insomnia [74], highlighting the therapeutic prospects of intervening in these molecular pathways. The findings from these studies imply that disturbances in host sleep can alter the expression of circadian rhythm-related genes, which then disrupt intestinal barrier integrity and impact the balance of gut microbiota. Conversely, disruptions in gut microbiota can impact sleep quality. A more profound comprehension of the interplay between these factors will contribute to the prevention and treatment of sleep disorders.

In addition to these genetic interactions, it is crucial to acknowledge the role of gut microbiota-derived metabolites, such as indoles, bile acids, and neurotransmitter precursors, in modulating circadian rhythms and sleep patterns. These metabolites act as signaling molecules interacting with host receptors, influencing the expression of circadian genes and thereby impacting sleep regulation.

Understanding the complex interplay among gut microbiota composition, circadian gene expression, and metabolite profiles provides a fertile ground for developing novel therapeutic strategies. Targeted interventions, including precision probiotics, dietary modifications designed to enhance beneficial metabolite production, and pharmacological agents that modulate specific circadian pathways, hold promise in restoring sleep-wake cycles and improving overall health in individuals with sleep disorders.

## 4 Gut microbiota interventions for sleep enhancement: Applications and challenges

The gut microbiota, through its integral role in the gut–brain axis, is a key player in preserving both physical and mental well-being. An increasing body of research on gut microbiota interventions, including the use of probiotics, prebiotics, postbiotics, and fecal microbiota transplantation (FMT), has illuminated the potential of modulating the gut microbiome as a novel therapeutic strategy for managing sleep disorders and enhancing overall sleep quality. This evolving understanding paves the way for innovative, microbial-based approaches to promote healthier sleep patterns.

### 4.1 Probiotics for sleep improvement

Probiotics, defined as live microorganisms conferring health benefits when administered in adequate amounts, have emerged as potential therapeutics for sleep enhancement. A double-blinded, placebo-controlled, crossover-designed trial showed that daily use of *Lactobacillus gasseri* CP2305 for 4 weeks showed significant improvements in anxiety, depression, sleep quality, and reduced cortisol levels, alongside alterations in gut microbiota composition [75], suggesting its potential benefits for managing stress-related behaviors. In another intervention by Lee et al., a probiotic mixture composed of *Limosilactobacillus reuteri* NK33 and *Bifidobacterium adolescentis* NK98 on 156 individuals with symptoms of anxiety and insomnia. The results demonstrated that this probiotic combination significantly enhanced sleep quality and notably reduced the abundance of Enterobacteriaceae in the gut [76], further supporting probiotics’ sleep-enhancing potential. A separate 17-week double-blind trial in rugby players found that the supplementation of *Saccharomyces boulardii* effectively enhances the sleep quality [77]. Another placebo-controlled, double-blind trial by Takada et al., demonstrated that the supplementation of *Lacticaseibacillus casei* Shirota ameliorated the sleep quality of individuals under stressful conditions [78]. These benefits microorganisms from their ability to modulate gut microbiota composition, increase bacterial diversity, and reduce pathogenic bacteria, thereby impacting the gut–brain axis positively.

While probiotics show promise in enhancing sleep, they encounter several pivotal challenges demanding attention. Chief among these is the issue of strain specificity, which underscores the need for meticulous research to discern the most efficacious strains for sleep therapy [79]. Individual variations in response, rooted in unique gut microbiomes and genetic backgrounds, introduce another layer of complexity, emphasizing the necessity for personalized strategies and complicating universal application. Lastly, the field is in want of extended longitudinal studies to ascertain the longevity of benefits and any potential adverse reactions over time, thereby filling a critical gap in our understanding of probiotics’ long-term implications for sleep health. Addressing these challenges is paramount to refine probiotic interventions, transforming them into a reliably effective and safely integrated part of sleep disorder management (Table 2).

**Table 2:** The studies summary of gut microbiota interventions for sleep enhancement

**Category**	**Type of intervention**	**Proposed mechanism**	**Principal findings**	**Reference**
**Probiotics**	*Lactobacillus gasseri* CP2305 on a double-blinded, placebo-controlled, crossover-designed trial	Inhibit the growth of harmful bacteria	Suppressed the growth of Enterobacteriaceae and effectively alleviated stress while improving sleep quality	[75]
	*Limosilactobacillus reuteri* NK33 and *Bifidobacterium adolescentis* NK98 on 156 individuals with symptoms of anxiety and insomnia	Regulate intestinal microecological balance and inhibit inflammatory responses	Significantly enhanced sleep quality and notably reduced the abundance of Enterobacteriaceae in the gut; decreased serum interleukin-6 levels; increased *Bifidobacteriaceae* and *Lactobacillacea*	[76]
	*Saccharomyces boulardii* on a 17-week double-blind trial on rugby players	Not reported	Effectively enhance the sleep quality of rugby players	[77]
	*Lacticaseibacillus casei* Shirota on a placebo-controlled double-blind trial	Not reported	improve the sleep quality of individuals under stressful conditions	[78]
**Prebiotics**	scGOS and lcFOS on sleep-deprived mice	Potentially by modulating inflammation levels and synchronizing circadian rhythms to foster optimal health	Increase in fecal SCFA levels. Reduction in sleep deprivation-induced inflammation and anxiety-like symptoms	[80]
	Lactoferrin and milk fat globule membrane in rat models.	Regulate intestinal microecological balance.	Enhanced gut microbiota diversity, potentially linked to improvements in sleep quality	[81]
	Polydextrose and galactooligosaccharides, a double-blind randomized trial in infants	Regulate intestinal microecological balance	Increase in nap duration	[82]
**Postbiotics**	5-HTP supplementation on sleep quality in 30 older adults over 12 weeks	Regulate intestinal microecological balance	Notably enhanced certain aspects of sleep, and led to increased gut microbiota diversity and SCFA-producing bacteria	[83]
	Heat-inactivated *L. gasseri* CP2305 on 60 Japanese medical students	Not reported	Reduced anxiety and sleep disturbance relative to placebo, attenuated the stress-induced decline of *Bifidobacterium* spp. and the stress-induced elevation of *Streptococcus* spp.	[84]
**FMT**	FMT in adults with chronic insomnia	Restore intestinal microecological balance.	Increase in genera like *Lactobacillus*, *Bifidobacterium*, and *Turicibacter* and a reduction in *Eggerthella*	[85]

### 4.2 Prebiotics and sleep regulation

Prebiotics, characterized by their resistance to human digestive enzymes while serving as nutrients for gut microorganisms, hold promise in enhancing sleep quality through their ability to shape microbial populations and metabolic outputs [86]. The study by Chung et al. demonstrated the positive effects of a prebiotic mixture of short-chain galactooligosaccharides and long-chain fructooligosaccharides (lcFOS) (9:1 ratio) on sleep-deprived mice. This regimen led to elevated fecal SCFA levels, which were concurrently associated with reduced inflammation and anxiety-like behaviors induced by sleep loss. These findings hint at a potential mechanism for the observed improvements in sleep [80]. Similarly, interventions involving lactoferrin and milk fat globule membrane in rat models exhibited enhanced gut microbiota diversity, potentially linked to improvements in sleep quality [81]. This augmentation in gut microbial diversity is increasingly recognized as a factor contributing to overall health, including aspects of sleep regulation. Moreover, a double-blind randomized trial in infants who received polydextrose and galactooligosaccharides supplementation reported an increase in nap duration [87]. This observation not only supports the role of prebiotics in influencing sleep patterns but also highlights their potential applicability across different age groups, indicating a versatile strategy for sleep health promotion.

**Figure 2:**
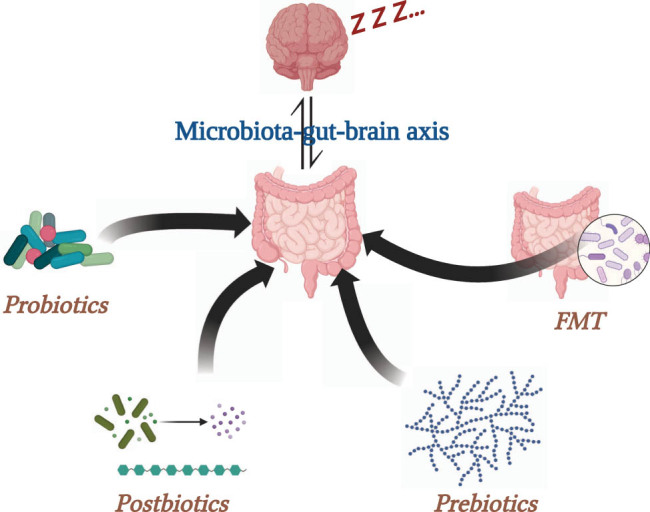
Proposed gut microbiota interventions to improve sleep quality. Four microbiota-targeted intervention approaches, including probiotics, prebiotics, postbiotics, and FMT, are depicted in the graph. These interventions have the potential to enhance sleep quality by modulating the microbiota–gut–brain axis, offering promising avenues for the treatment of sleep disorders.

Prebiotic use for sleep regulation encounters challenges including the need for individualized dosing strategies, given varying responses tied to unique microbiome compositions. Long-term efficacy and safety profiles require further investigation to ensure sustainable benefits without adverse effects. Standardization issues persist due to a lack of consensus on defining and classifying prebiotics, complicating research comparability. Personalized approaches, accounting for individual gut microbiota variations, are imperative for maximizing benefits (Figure 2).

### 4.3 Postbiotics: a novel avenue for sleep enhancement

Postbiotics are metabolic products of bacteria or fragments of their cell bodies that can provide physiological benefits to the host while avoiding the risks associated with live microorganisms, such as infections. A single-blinded, randomized controlled trial examined the effects of 5-HTP supplementation on sleep quality and gut microbiota in older adults over 12 weeks, revealing that 5-HTP notably enhanced certain aspects of sleep, particularly in individuals with initially poor sleep quality, and led to increased gut microbiota diversity and SCFA-producing bacteria [83], suggesting a potential role in sleep improvement and gut health modulation. Moreover, long-term supplementation with heat-inactivated *L. gasseri* CP2305 in healthy adults reduced anxiety, improved sleep quality, and positively modulated gut microbiota, indicating potential benefits for mental health and sleep under stress.

The exploration of postbiotics for sleep enhancement faces a scarcity of studies, demanding expanded research to bolster evidence. Elucidating the exact mechanisms and identifying specific bioactive compounds are imperative. Standardized approaches for postbiotic characterization and quantification remain underdeveloped, posing obstacles to consistency and comparison across investigations.

### 4.4 FMT in sleep health

FMT is a therapeutic approach in which the entire microbiota from a donor is transplanted into a recipient to restore the recipient’s gut microbiota balance, which is primarily used to treat recurrent *Clostridium difficile* infections. However, recent exploratory studies have ventured into uncharted territories, assessing its potential in managing conditions beyond gastrointestinal disorders, including sleep health [88]. A study assessed the efficacy and safety of FMT in adults with chronic insomnia, comparing outcomes to a non-insomnia group. Results showed significant improvements in insomnia symptoms, anxiety, depression, and quality of life for insomnia patients post-FMT, with 76.47% achieving primary endpoints. The gut microbiota changes, specifically an increase in genera like *Lactobacillus*, *Bifidobacterium*, *Turicibacter*, and a reduction in *Eggerthella*, correlated with FMT effectiveness [85], suggesting FMT as a potential novel treatment for chronic insomnia with implications for sleep quality improvement.

Implementing FMT for sleep health faces numerous challenges, including ethical concerns around donor screening, standardization of procedures, and ensuring long-term safety and efficacy. Future research is expected to delve deeper into the mechanistic understanding of how FMT influences sleep, focusing on the specific microbial species and metabolites involved. Personalized FMT strategies, based on an individual’s microbiome profile, may emerge as a future direction.

Sleep disorder treatments conventionally involve drugs, psychotherapy, and traditional Chinese medicine, overlooking microbiota interventions. However, advances in understanding the gut microbiota-sleep link have sparked exploration into microbiota-targeted therapies for sleep enhancement. Personalized sleep therapy via gut microbiota modulation, using probiotics, prebiotics, and tailored interventions based on individual microbiota profiles, emerges as a promising frontier, prioritizing metabolite research for systemic effects.

## 5 Conclusions and perspectives

Emerging research highlights the intricate relationship between the gut microbiota and sleep, primarily orchestrated through the gut–brain axis that connects these two vital components of our physiology. While sleep disturbances can markedly alter the gut microbiota’s composition and function, the heterogeneity observed in these effects across studies underscores the need for further exploration of individual variability, species-specific responses, and the myriad environmental and intrinsic factors influencing gut microbial homeostasis. Promisingly, targeted interventions, including specific prebiotics such as polydextrose and galactooligosaccharides, probiotics like *Bifidobacterium* and *Lactobacillus* strains, and postbiotics like butyrate, have demonstrated potential in ameliorating gut dysbiosis and enhancing sleep quality. Mechanistically, these interventions may influence sleep by modulating neurotransmitter production, reducing inflammation, and stabilizing circadian rhythms.

Despite these advancements, it is imperative to recognize the existing limitations in our understanding, which include the lack of large-scale, longitudinal studies elucidating causality, and the complexity in deciphering the direct versus indirect effects of gut microbiota on sleep regulation. Future research should aim to clarify the precise mechanisms underlying the gut microbiota’s impact on sleep, identify biomarkers predictive of sleep disturbances, and evaluate the long-term efficacy and safety of microbiota-targeted interventions in diverse populations.

In conclusion, the reciprocal relationship between sleep and the gut microbiota opens new vistas for preventive and therapeutic strategies targeting sleep disorders. However, whether gut microbiota dysbiosis acts as a causative agent in sleep disorders or serves as an indirect contributor remains a subject demanding further in-depth investigation. The complex interplay between sleep and the gut microbiota warrants continued research efforts to fully decipher its far-reaching implications on human health and overall wellness.
